# Real World Problem-Solving

**DOI:** 10.3389/fnhum.2018.00261

**Published:** 2018-06-26

**Authors:** Vasanth Sarathy

**Affiliations:** Human-Robot Interaction Laboratory, Department of Computer Science, Tufts University, Medford, MA, United States

**Keywords:** creativity, problem-solving, insight, attention network, salience network, default mode network

## Abstract

Real world problem-solving (RWPS) is what we do every day. It requires flexibility, resilience, resourcefulness, and a certain degree of creativity. A crucial feature of RWPS is that it involves continuous interaction with the environment during the problem-solving process. In this process, the environment can be seen as not only a source of inspiration for new ideas but also as a tool to facilitate creative thinking. The cognitive neuroscience literature in creativity and problem-solving is extensive, but it has largely focused on neural networks that are active when subjects are *not* focused on the outside world, i.e., not using their environment. In this paper, I attempt to combine the relevant literature on creativity and problem-solving with the scattered and nascent work in perceptually-driven learning from the environment. I present my synthesis as a potential new theory for real world problem-solving and map out its hypothesized neural basis. I outline some testable predictions made by the model and provide some considerations and ideas for experimental paradigms that could be used to evaluate the model more thoroughly.

## 1. Introduction

In the Apollo 13 space mission, astronauts together with ground control had to overcome several challenges to bring the team safely back to Earth (Lovell and Kluger, 2006). One of these challenges was controlling carbon dioxide levels onboard the space craft: “For 2 days straight [they] had worked on how to jury-rig the Odysseys canisters to the Aquarius's life support system. Now, using materials known to be available onboard the spacecraft—a sock, a plastic bag, the cover of a flight manual, lots of duct tape, and so on—the crew assembled a strange contraption and taped it into place. Carbon dioxide levels immediately began to fall into the safe range” (Team, 1970; Cass, 2005).

The success of Apollo 13's recovery from failure is often cited as a glowing example of human resourcefulness and inventiveness alongside more well-known inventions and innovations over the course of human history. However, this sort of inventive capability is not restricted to a few creative geniuses, but an ability present in all of us, and exemplified in the following mundane example. Consider a situation when your only suit is covered in lint and you do not own a lint remover. You see a roll of duct tape, and being resourceful you reason that it might be a good substitute. You then solve the problem of lint removal by peeling a full turn's worth of tape and re-attaching it backwards onto the roll to expose the sticky side all around the roll. By rolling it over your suit, you can now pick up all the lint.

In both these examples (historic as well as everyday), we see evidence for our innate ability to problem-solve in the real world. Solving real world problems in real time given constraints posed by one's environment are crucial for survival. At the core of this skill is our mental capability to get out of “sticky situations” or impasses, i.e., difficulties that appear unexpectedly as impassable roadblocks to solving the problem at hand. But, what are the cognitive processes that enable a problem solver to overcome such impasses and arrive at a solution, or at least a set of promising next steps?

A central aspect of this type of real world problem solving, is the role played by the solver's surrounding environment during the problem-solving process. Is it possible that interaction with one's environment can facilitate creative thinking? The answer to this question seems somewhat obvious when one considers the most famous anecdotal account of creative problem solving, namely that of Archimedes of Syracuse. During a bath, he found a novel way to check if the King's crown contained non-gold impurities. The story has traditionally been associated with the so-called “Eureka moment,” the sudden affective experience when a solution to a particularly thorny problem emerges. In this paper, I want to temporarily turn our attention away from the specific “aha!” experience itself and take particular note that Archimedes made this discovery, not with his eyes closed at a desk, but in a real-world context of a bath^1^. The bath was not only a passive, relaxing environment for Archimedes, but also a specific source of inspiration. Indeed it was his noticing the displacement of water that gave him a specific methodology for measuring the purity of the crown; by comparing how much water a solid gold bar of the same weight would displace as compared with the crown. This sort of continuous environmental interaction was present when the Apollo 13 engineers discovered their life-saving solution, and when you solved the suit-lint-removal problem with duct tape.

The neural mechanisms underlying problem-solving have been extensively studied in the literature, and there is general agreement about the key functional networks and nodes involved in various stages of problem-solving. In addition, there has been a great deal of work in studying the neural basis for creativity and insight problem solving, which is associated with the sudden emergence of solutions. However, in the context of problem-solving, creativity, and insight have been researched as largely an internal process without much interaction with and influence from the external environment (Wegbreit et al., 2012; Abraham, 2013; Kounios and Beeman, 2014)^2^. Thus, there are open questions of what role the environment plays during real world problem-solving (RWPS) and how the brain enables the assimilation of novel items during these external interactions.

In this paper, I synthesize the literature on problem-solving, creativity and insight, and particularly focus on how the environment can inform RWPS. I explore three environmentally-informed mechanisms that could play a critical role: (1) partial-cue driven context-shifting, (2) heuristic prototyping and learning novel associations, and (3) learning novel physical inferences. I begin first with some intuitions about real world problem solving, that might help ground this discussion and providing some key distinctions from more traditional problem solving research. Then, I turn to a review of the relevant literature on problem-solving, creativity, and insight first, before discussing the three above-mentioned environmentally-driven mechanisms. I conclude with a potential new model and map out its hypothesized neural basis.

## 2. Problem solving, creativity, and insight

### 2.1. What is real world problem-solving?

Archimedes was embodied in the real world when he found his solution. In fact, the real world helped him solve the problem. Whether or not these sorts of historic accounts of creative inspiration are accurate^3^, they do correlate with some of our own key intuitions about how problem solving occurs “in the wild.” Real world problem solving (RWPS) is different from those that occur in a classroom or in a laboratory during an experiment. They are often dynamic and discontinuous, accompanied by many starts and stops. Solvers are never working on just one problem. Instead, they are simultaneously juggling several problems of varying difficulties and alternating their attention between them. Real world problems are typically ill-defined, and even when they are well-defined, often have open-ended solutions. Coupled with that is the added aspect of uncertainty associated with the solver's problem solving strategies. As introduced earlier, an important dimension of RWPS is the continuous interaction between the solver and their environment. During these interactions, the solver might be inspired or arrive at an “aha!” moment. However, more often than not, the solver experiences dozens of minor discovery events— “hmmm, interesting…” or “wait, what?…” moments. Like discovery events, there's typically never one singular impasse or distraction event. The solver must iterate through the problem solving process experiencing and managing these sorts of intervening events (including impasses and discoveries). In summary, RWPS is quite messy and involves a tight interplay between problem solving, creativity, and insight. Next, I explore each of these processes in more detail and explicate a possible role of memory, attention, conflict management and perception.

### 2.2. Analytical problem-solving

In psychology and neuroscience, *problem-solving* broadly refers to the inferential steps taken by an agent^4^ that leads from a given state of affairs to a desired goal state (Barbey and Barsalou, 2009). The agent does not immediately know how this goal can be reached and must perform some mental operations (i.e., thinking) to determine a solution (Duncker, 1945).

The problem solving literature divides problems based on clarity (well-defined vs. ill-defined) or on the underlying cognitive processes (analytical, memory retrieval, and insight) (Sprugnoli et al., 2017). While memory retrieval is an important process, I consider it as a sub-process to problem solving more generally. I first focus on analytical problem-solving process, which typically involves problem-representation and encoding, and the process of forming and executing a solution plan (Robertson, 2016).

#### 2.2.1. Problem definition and representation

An important initial phase of problem-solving involves defining the problem and forming a representation in the working memory. During this phase, components of the prefrontal cortex (PFC), default mode network (DMN), and the dorsal anterior cingulate cortex (dACC) have been found to be activated. If the problem is familiar and well-structured, top-down executive control mechanisms are engaged and the left prefrontal cortex including the frontopolar, dorso-lateral (dlPFC), and ventro-lateral (vlPFC) are activated (Barbey and Barsalou, 2009). The DMN along with the various structures in the medial temporal lobe (MTL) including the hippocampus (HF), parahippocampal cortex, perirhinal and entorhinal cortices are also believed to have limited involvement, especially in episodic memory retrieval activities during this phase (Beaty et al., 2016). The problem representation requires encoding problem information for which certain visual and parietal areas are also involved, although the extent of their involvement is less clear (Anderson and Fincham, 2014; Anderson et al., 2014).

##### 2.2.1.1. Working memory

An important aspect of problem representation is the engagement and use of working memory (WM). The WM allows for the maintenance of relevant problem information and description in the mind (Gazzaley and Nobre, 2012). Research has shown that WM tasks consistently recruit the dlPFC and left inferior frontal cortex (IC) for encoding an manipulating information; dACC for error detection and performance adjustment; and vlPFC and the anterior insula (AI) for retrieving, selecting information and inhibitory control (Chung and Weyandt, 2014; Fang et al., 2016).

##### 2.2.1.2. Representation

While we generally have a sense for the brain regions that are functionally influential in problem definition, less is known about how exactly events are represented *within* these regions. One theory for how events are represented in the PFC is the structured event complex theory (SEC), in which components of the event knowledge are represented by increasingly higher-order convergence zones localized within the PFC, akin to the convergence zones (from posterior to anterior) that integrate sensory information in the brain (Barbey et al., 2009). Under this theory, different zones in the PFC (left vs. right, anterior vs. posterior, lateral vs. medial, and dorsal vs. ventral) represent different aspects of the information contained in the events (e.g., number of events to be integrated together, the complexity of the event, whether planning, and action is needed). Other studies have also suggested the CEN's role in tasks requiring cognitive flexibility, and functions to switch thinking modes, levels of abstraction of thought and consider multiple concepts simultaneously (Miyake et al., 2000).

Thus, when the problem is well-structured, problem representation is largely an executive control activity coordinated by the PFC in which problem information from memory populates WM in a potentially structured representation. Once the problem is defined and encoded, planning and execution of a solution can begin.

#### 2.2.2. Planning

The central executive network (CEN), particularly the PFC, is largely involved in plan formation and in plan execution. Planning is the process of generating a strategy to advance from the current state to a goal state. This in turn involves retrieving a suitable solution strategy from memory and then coordinating its execution.

##### 2.2.2.1. Plan formation

The dlPFC supports sequential planning and plan formation, which includes the generation of hypothesis and construction of plan steps (Barbey and Barsalou, 2009). Interestingly, the vlPFC and the angular gyrus (AG), implicated in a variety of functions including memory retrieval, are also involved in plan formation (Anderson et al., 2014). Indeed, the AG together with the regions in the MTL (including the HF) and several other regions form a what is known as the “core” network. The core network is believed to be activated when recalling past experiences, imagining fictitious, and future events and navigating large-scale spaces (Summerfield et al., 2010), all key functions for generating plan hypotheses. A recent study suggests that the AG is critical to both episodic simulation, representation, and episodic memory (Thakral et al., 2017). One possibility for how plans are formulated could involve a dynamic process of retrieving an optimal strategy from memory. Research has shown significant interaction between striatal and frontal regions (Scimeca and Badre, 2012; Horner et al., 2015). The striatum is believed to play a key role in declarative memory retrieval, and specifically helping retrieve *optimal* (or previously rewarded) memories (Scimeca and Badre, 2012). Relevant to planning and plan formation, Scimeca & Badre have suggested that the striatum plays two important roles: (1) in mapping acquired value/utility to action selection, and thereby helping plan formation, and (2) modulation and re-encoding of actions and other plan parameters. Different types of problems require different sets of specialized knowledge. For example, the knowledge needed to solve mathematical problems might be quite different (albeit overlapping) from the knowledge needed to select appropriate tools in the environment.

Thus far, I have discussed planning and problem representation as being domain-independent, which has allowed me to outline key areas of the PFC, MTL, and other regions relevant to all problem-solving. However, some types of problems require domain-specific knowledge for which other regions might need to be recruited. For example, when planning for tool-use, the superior parietal lobe (SPL), supramarginal gyrus (SMG), anterior inferior parietal lobe (AIPL), and certain portions of the temporal and occipital lobe involved in visual and spatial integration have been found to be recruited (Brandi et al., 2014). It is believed that domain-specific information stored in these regions is recovered and used for planning.

##### 2.2.2.2. Plan execution

Once a solution plan has been recruited from memory and suitably tuned for the problem on hand, the left-rostral PFC, caudate nucleus (CN), and bilateral posterior parietal cortices (PPC) are responsible for translating the plan into executable form (Stocco et al., 2012). The PPC stores and maintains “mental template” of the executable form. Hemispherical division of labor is particularly relevant in planning where it was shown that when planning to solve a Tower of Hanoi (block moving) problem, the right PFC is involved in plan construction whereas the left PFC is involved in controlling processes necessary to supervise the execution of the plan (Newman and Green, 2015). On a separate note and not the focus of this paper, plan execution and problem-solving can require the recruitment of affective and motivational processing in order to supply the agent with the resolve to solve problems, and the vmPFC has been found to be involved in coordinating this process (Barbey and Barsalou, 2009).

### 2.3. Creativity

During the gestalt movement in the 1930s, Maier noted that “most instances of “real” problem solving involves creative thinking” (Maier, 1930). Maier performed several experiments to study mental fixation and insight problem solving. This close tie between insight and creativity continues to be a recurring theme, one that will be central to the current discussion. If creativity and insight are linked to RWPS as noted by Maier, then it is reasonable to turn to the creativity and insight literature for understanding the role played by the environment. A large portion of the creativity literature has focused on viewing creativity as an internal process, one in which the solvers attention is directed inwards, and toward internal stimuli, to facilitate the generation of novel ideas and associations in memory (Beaty et al., 2016). Focusing on imagination, a number of researchers have looked at blinking, eye fixation, closing eyes, and looking nowhere behavior and suggested that there is a shift of attention from external to internal stimuli during creative problem solving (Salvi and Bowden, 2016). The idea is that shutting down external stimuli reduces cognitive load and focuses attention internally. Other experiments studying sleep behavior have also noted the beneficial role of internal stimuli in problem solving. The notion of ideas popping into ones consciousness, suddenly, during a shower is highly intuitive for many and researchers have attempted to study this phenomena through the lens of incubation, and unconscious thought that is internally-driven. There have been several theories and counter-theories proposed to account specifically for the cognitive processes underlying incubation (Ritter and Dijksterhuis, 2014; Gilhooly, 2016), but none of these theories specifically address the role of the external environment.

The neuroscience of creativity has also been extensively studied and I do not focus on an exhaustive literature review in this paper (a nice review can be found in Sawyer, 2011). From a problem-solving perspective, it has been found that unlike well-structured problems, ill-structured problems activate the right dlPFC. Most of the past work on creativity and creative problem-solving has focused on exploring memory structures and performing internally-directed searches. Creative idea generation has primarily been viewed as internally directed attention (Jauk et al., 2012; Benedek et al., 2016) and a primary mechanism involved is *divergent thinking*, which is the ability to produce a variety of responses in a given situation (Guilford, 1962). Divergent thinking is generally thought to involve interactions between the DMN, CEN, and the salience network (Yoruk and Runco, 2014; Heinonen et al., 2016). One psychological model of creative cognition is the Geneplore model that considers two major phases of generation (memory retrieval and mental synthesis) and exploration (conceptual interpretation and functional inference) (Finke et al., 1992; Boccia et al., 2015). It has been suggested that the associative mode of processing to generate new creative association is supported by the DMN, which includes the medial PFC, posterior cingulate cortex (PCC), tempororparietal juntion (TPJ), MTL, and IPC (Beaty et al., 2014, 2016).

That said, the creativity literature is not completely devoid of acknowledging the role of the environment. In fact, it is quite the opposite. Researchers have looked closely at the role played by externally provided hints from the time of the early gestalt psychologists and through to present day studies (Öllinger et al., 2017). In addition to studying how hints can help problem solving, researchers have also looked at how directed action can influence subsequent problem solving—e.g., swinging arms prior to solving the two-string puzzle, which requires swinging the string (Thomas and Lleras, 2009). There have also been numerous studies looking at how certain external perceptual cues are correlated with creativity measures. Vohs et al. suggested that untidiness in the environment and the increased number of potential distractions helps with creativity (Vohs et al., 2013). Certain colors such as blue have been shown to help with creativity and attention to detail (Mehta and Zhu, 2009). Even environmental illumination, or lack thereof, have been shown to promote creativity (Steidle and Werth, 2013). However, it is important to note that while these and the substantial body of similar literature show the relationship of the environment to creative problem solving, they do not specifically account for the cognitive processes underlying the RWPS when external stimuli are received.

### 2.4. Insight problem solving

Analytical problem solving is believed to involve deliberate and conscious processing that advances step by step, allowing solvers to be able to explain exactly how they solved it. Inability to solve these problems is often associated with lack of required prior knowledge, which if provided, immediately makes the solution tractable. Insight, on the other hand, is believed to involve a sudden and unexpected emergence of an obvious solution or strategy sometimes accompanied by an affective aha! experience. Solvers find it difficult to consciously explain how they generated a solution in a sequential manner. That said, research has shown that having an aha! moment is neither necessary nor sufficient to insight and vice versa (Danek et al., 2016). Generally, it is believed that insight solvers acquire a full and deep understanding of the problem when they have solved it (Chu and Macgregor, 2011). There has been an active debate in the problem solving community about whether insight is something special. Some have argued that it is not, and that there are no special or spontaneous processes, but simply a good old-fashioned search of a large problem space (Kaplan and Simon, 1990; MacGregor et al., 2001; Ash and Wiley, 2006; Fleck, 2008). Others have argued that insight is special and suggested that it is likely a different process (Duncker, 1945; Metcalfe, 1986; Kounios and Beeman, 2014). This debate lead to two theories for insight problem solving. MacGregor et al. proposed the Criterion for Satisfactory Progress Theory (CSPT), which is based on Newell and Simons original notion of problem solving as being a heuristic search through the problem space (MacGregor et al., 2001). The key aspect of CSPT is that the solver is continually monitoring their progress with some set of criteria. Impasses arise when there is a criterion failure, at which point the solver tries non-maximal but promising states. The representational change theory (RCT) proposed by Ohlsson et al., on the other hand, suggests that impasses occur when the goal state is not reachable from an initial problem representation (which may have been generated through unconscious spreading activation) (Ohlsson, 1992). In order to overcome an impasse, the solver needs to restructure the problem representation, which they can do by (1) elaboration (noticing new features of a problem), (2) re-encoding fixing mistaken or incomplete representations of the problem, and by (3) changing constraints. Changing constraints is believed to involve two sub-processes of constraint relaxation and chunk-decomposition.

The current position is that these two theories do not compete with each other, but instead complement each other by addressing different stages of problem solving: pre- and post-impasse. Along these lines, Ollinger et al. proposed an extended RCT (eRCT) in which revising the search space and using heuristics was suggested as being a dynamic and iterative and recursive process that involves repeated instances of search, impasse and representational change (Öllinger et al., 2014, 2017). Under this theory, a solver first forms a problem representation and begins searching for solutions, presumably using analytical problem solving processes as described earlier. When a solution cannot be found, the solver encounters an impasse, at which point the solver must restructure or change the problem representation and once again search for a solution. The model combines both analytical problem solving (through heuristic searches, hill climbing and progress monitoring), and creative mechanisms of constraint relaxation and chunk decomposition to enable restructuring.

Ollingers model appears to comprehensively account for both analytical and insight problem solving and, therefore, could be a strong candidate to model RWPS. However, while compelling, it is nevertheless an insufficient model of RWPS for many reasons, of which two are particularly significant for the current paper. First, the model does explicitly address mechanisms by which external stimuli might be assimilated. Second, the model is not sufficiently flexible to account for other events (beyond impasse) occurring during problem solving, such as distraction, mind-wandering and the like.

So, where does this leave us? I have shown the interplay between problem solving, creativity and insight. In particular, using Ollinger's proposal, I have suggested (maybe not quite explicitly up until now) that RWPS involves some degree of analytical problem solving as well as the post-impasse more creative modes of problem restructuring. I have also suggested that this model might need to be extended for RWPS along two dimensions. First, events such as impasses might just be an instance of a larger class of events that intervene during problem solving. Thus, there needs to be an accounting of the cognitive mechanisms that are potentially influenced by impasses and these other intervening events. It is possible that these sorts of events are crucial and trigger a switch in attentional focus, which in turn facilitates switching between different problem solving modes. Second, we need to consider when and how externally-triggered stimuli from the solver's environment can influence the problem solving process. I detail three different mechanisms by which external knowledge might influence problem solving. I address each of these ideas in more detail in the next two sections.

## 3. Event-triggered mode switching during problem-solving

### 3.1. Impasse

When solving certain types of problems, the agent might encounter an impasse, i.e., some block in its ability to solve the problem (Sprugnoli et al., 2017). The impasse may arise because the problem may have been ill-defined to begin with causing incomplete and unduly constrained representations to have been formed. Alternatively, impasses can occur when suitable solution strategies cannot be retrieved from memory or fail on execution. In certain instances, the solution strategies may not exist and may need to be generated from scratch. Regardless of the reason, an impasse is an interruption in the problem solving process; one that was running conflict-free up until the point when a seemingly unresolvable issue or an error in the predicted solution path was encountered. Seen as a conflict encountered in the problem-solving process it activates the anterior cingulate cortex (ACC). It is believed that the ACC not only helps detect the conflict, but also switch modes from one of “exploitation” (planning) to “exploration” (search) (Quilodran et al., 2008; Tang et al., 2012), and monitors progress during resolution (Chu and Macgregor, 2011). Some mode switching duties are also found to be shared with the AI (the ACC's partner in the salience network), however, it is unclear exactly the extent of this function-sharing.

Even though it is debatable if impasses are a necessary component of insight, they are still important as they provide a starting point for the creativity (Sprugnoli et al., 2017). Indeed, it is possible that around the moment of impasse, the AI and ACC together, as part of the salience network play a crucial role in switching thought modes from analytical planning mode to creative search and discovery mode. In the latter mode, various creative mechanisms might be activated allowing for a solution plan to emerge. Sowden et al. and many others have suggested that the salience network is potentially a candidate neurobiological mechanism for shifting between thinking processes, more generally (Sowden et al., 2015). When discussing various dual-process models as they relate to creative cognition, Sowden et al. have even noted that the ACC activation could be useful marker to identify shifting as participants work creative problems.

### 3.2. Defocused attention

As noted earlier, in the presence of an impasse there is a shift from an exploitative (analytical) thinking mode to an exploratory (creative) thinking mode. This shift impacts several networks including, for example, the attention network. It is believed attention can switch between a focused mode and a defocused mode. Focused attention facilitates analytic thought by constraining activation such that items are considered in a compact form that is amenable to complex mental operations. In the defocused mode, agents expand their attention allowing new associations to be considered. Sowden et al. (2015) note that the mechanism responsible for adjustments in cognitive control may be linked to the mechanisms responsible for attentional focus. The generally agreed position is that during generative thinking, unconscious cognitive processes activated through defocused attention are more prevalent, whereas during exploratory thinking, controlled cognition activated by focused attention becomes more prevalent (Kaufman, 2011; Sowden et al., 2015).

Defocused attention allows agents to not only process different aspects of a situation, but to also activate additional neural structures in long term memory and find new associations (Mendelsohn, 1976; Yoruk and Runco, 2014). It is believed that cognitive material attended to and cued by positive affective state results in defocused attention, allowing for more complex cognitive contexts and therefore a greater range of interpretation and integration of information (Isen et al., 1987). High attentional levels are commonly considered a typical feature of highly creative subjects (Sprugnoli et al., 2017).

## 4. Role of the environment

In much of the past work the focus has been on treating creativity as largely an internal process engaging the DMN to assist in making novel connections in memory. The suggestion has been that “individual needs to suppress external stimuli and concentrate on the inner creative process during idea generation” (Heinonen et al., 2016). These ideas can then function as seeds for testing and problem-solving. While true of many creative acts, this characterization does not capture how creative ideas arise in many real-world creative problems. In these types of problems, the agent is functioning and interacting with its environment before, during and after problem-solving. It is natural then to expect that stimuli from the environment might play a role in problem-solving. More specifically, it can be expected that through passive and active involvement with the environment, the agent is (1) able to trigger an unrelated, but potentially useful memory relevant for problem-solving, (2) make novel connections between two events in memory with the environmental cue serving as the missing link, and (3) incorporate a completely novel information from events occuring in the environment directly into the problem-solving process. I explore potential neural mechanisms for these three types of environmentally informed creative cognition, which I hypothesize are enabled by defocused attention.

### 4.1. Partial cues trigger relevant memories through context-shifting

I have previously discussed the interaction between the MTL and PFC in helping select task-relevant and critical memories for problem-solving. It is well-known that pattern completion is an important function of the MTL and one that enables memory retrieval. Complementary Learning Theory (CLS) and its recently updated version suggest that the MTL and related structures support initial storage as well as retrieval of item and context-specific information (Kumaran et al., 2016). According to CLS theory, the dentate gyrus (DG) and the CA3 regions of the HF are critical to selecting neural activity patterns that correspond to particular experiences (Kumaran et al., 2016). These patterns might be distinct even if experiences are similar and are stabilized through increases in connection strengths between the DG and CA3. Crucially, because of the connection strengths, reactivation of part of the pattern can activate the rest of it (i.e., pattern completion). Kumaran et al. have further noted that if consistent with existing knowledge, these new experiences can be quickly replayed and interleaved into structured representations that form part of the semantic memory.

Cues in the environment provided by these experiences hold partial information about past stimuli or events and this partial information converges in the MTL. CLS accounts for how these cues might serve to reactivate partial patterns, thereby triggering pattern completion. When attention is defocused I hypothesize that (1) previously unnoticed partial cues are considered, and (2) previously noticed partial cues are decomposed to produce previously unnoticed sub-cues, which in turn are considered. Zabelina et al. (2016) have shown that real-world creativity and creative achievement is associated with “leaky attention,” i.e., attention that allows for irrelevant information to be noticed. In two experiments they systematically explored the relationship between two notions of creativity— divergent thinking and real-world creative achievement—and the use of attention. They found that attentional use is associated in different ways for each of the two notions of creativity. While divergent thinking was associated with flexible attention, it does not appear to be leaky. Instead, selective focus and inhibition components of attention were likely facilitating successful performance on divergent thinking tasks. On the other hand, real-world creative achievement was linked to leaky attention. RWPS involves elements of both divergent thinking and of real-world creative achievement, thus I would expect some amount of attentional leaks to be part of the problem solving process.

Thus, it might be the case that a new set of cues or sub-cues “leak” in and activate memories that may not have been previously considered. These cues serve to reactivate a diverse set of patterns that then enable accessing a wide range of memories. Some of these memories are extra-contextual, in that they consider the newly noticed cues in several contexts. For example, when unable to find a screwdriver, we might consider using a coin. It is possible that defocused attention allows us to consider the coin's edge as being a potentially relevant cue that triggers uses for the thin edge outside of its current context in a coin. The new cues (or contexts) may allow new associations to emerge with cues stored in memory, which can occur during incubation. Objects and contexts are integrated into memory automatically into a blended representation and changing contexts disrupts this recognition (Hayes et al., 2007; Gabora, 2016). Cue-triggered context shifting allows an agent to break-apart a memory representation, which can then facilitate problem-solving in new ways.

### 4.2. Heuristic prototyping facilitates novel associations

It has long been the case that many scientific innovations have been inspired by events in nature and the surrounding environment. As noted earlier, Archimedes realized the relationship between the volume of an irregularly shaped object and the volume of water it displaced. This is an example of heuristic prototyping where the problem-solver notices an event in the environment, which then triggers the automatic activation of a heuristic prototype and the formation of novel associations (between the function of the prototype and the problem) which they can then use to solve the problem (Luo et al., 2013). Although still in its relative infancy, there has been some recent research into the neural basis for heuristic prototyping. Heuristic prototype has generally been defined as an enlightening prototype event with a similar element to the current problem and is often composed of a feature and a function (Hao et al., 2013). For example, in designing a faster and more efficient submarine hull, a heuristic prototype might be a shark's skin, while an unrelated prototype might be a fisheye camera (Dandan et al., 2013).

Research has shown that activating the feature function of the right heuristic prototype and linking it by way of semantic similarity to the required function of the problem was the key mechanism people used to solve several scienitific insight problems (Yang et al., 2016). A key region activated during heuristic prototyping is the dlPFC and it is believed to be generally responsible for encoding the events into memory and may play an important role in selecting and retrieving the matched unsolved technical problem from memory (Dandan et al., 2013). It is also believed that the precuneus plays a role in automatic retrieval of heuristic information allowing the heuristic prototype and the problem to combine (Luo et al., 2013). In addition to semantic processing, certain aspects of visual imagery have also been implicated in heuristic prototyping leading to the suggestion of the involvement of Broadman's area BA 19 in the occipital cortex.

There is some degree of overlap between the notions of heuristic prototyping and analogical transfer (the mapping of relations from one domain to another). Analogical transfer is believed to activate regions in the left medial fronto-parietal system (dlPFC and the PPC) (Barbey and Barsalou, 2009). I suggest here that analogical reasoning is largely an internally-guided process that is aided by heuristic prototyping which is an externally-guided process. One possible way this could work is if heuristic prototyping mechanisms help locate the relevant memory with which to then subsequently analogize.

### 4.3. Making physical inferences to acquire novel information

The agent might also be able to learn novel facts about their environment through passive observation as well as active experimentation. There has been some research into the neural basis for causal reasoning (Barbey and Barsalou, 2009; Operskalski and Barbey, 2016), but beyond its generally distributed nature, we do not know too much more. Beyond abstract causal reasoning, some studies looked into the cortical regions that are activated when people watch and predict physical events unfolding in real-time and in the real-world (Fischer et al., 2016). It was found that certain regions were associated with representing types of physical concepts, with the left intraparietal sulcus (IPS) and left middle frontal gyrus (MFG) shown to play a role in attributing causality when viewing colliding objects (Mason and Just, 2013). The parahippocampus (PHC) was associated with linking causal theory to observed data and the TPJ was involved in visualizing movement of objects and actions in space (Mason and Just, 2013).

## 5. Proposed theory

I noted earlier that Ollinger's model for insight problem solving, while serving as a good candidate for RWPS, requires extension. In this section, I propose a candidate model that includes some necessary extensions to Ollinger's framework. I begin by laying out some preliminary notions that underlie the proposed model.

### 5.1. Dual attentional modes

I propose that the attention-switching mechanism described earlier is at the heart of RWPS and enables two modes of operation: focused and defocused mode. In the focused mode, the problem representation is more or less fixed, and problem solving proceeds in a focused and goal directed manner through search, planning, and execution mechanisms. In the defocused mode, problem solving is not necessarily goal directed, but attempts to generate ideas, driven by both internal and external items.

At first glance, these modes might seem similar to convergent and divergent thinking modes postulated by numerous others to account for creative problem solving. Divergent thinking allows for the generation of new ideas and convergent thinking allows for verification and selection of generated ideas. So, it might seem that focused mode and convergent thinking are similar and likewise divergent and defocused mode. They are, however, quite different. The modes relate less to idea generation and verification, and more to the specific mechanisms that are operating with regard to a particular problem at a particular moment in time. Convergent and divergent processes may be occurring during both defocused and focused modes. Some degree of divergent processes may be used to search and identify specific solution strategies in focused mode. Also, there might be some degree of convergent idea verification occuring in defocused mode as candidate items are evaluated for their fit with the problem and goal. Thus, convergent and divergent thinking are one amongst many mechanisms that are utilized in focused and defocused mode. Each of these two modes has to do with degree of attention placed on a particular problem.

There have been numerous dual-process and dual-systems models of cognition proposed over the years. To address criticisms raised against these models and to unify some of the terminology, Evans & Stanovich proposed a dual-process model comprising Type 1 and Type 2 thought (Evans and Stanovich, 2013; Sowden et al., 2015). Type 1 processes are those that are believed to be autonomous and do not require working memory. Type 2 processes, on the other hand, are believed to require working memory and are cognitively decoupled to prevent real-world representations from becoming confused with mental simulations (Sowden et al., 2015). While acknowledging various other attributes that are often used to describe dual process models (e.g., fast/slow, associative/rule-based, automatic/controlled), Evans & Stanovich note that these attributes are merely frequent correlates and not defining characteristics of Type 1 or Type 2 processes. The proposed dual attentional modes share some similarities with the Evans & Stanovich Type 1 and 2 models. Specifically, Type 2 processes might occur in focused attentional mode in the proposed model as they typically involve the working memory and certain amount of analytical thought and planning. Similarly, Type 1 processes are likely engaged in defocused attentional mode as there are notions of associative and generative thinking that might be facilitated when attention has been defocused. The crucial difference between the proposed model and other dual-process models is that the dividing line between focused and defocused attentional modes is the degree of openness to internal and external stimuli (by various networks and functional units in the brain) when problem solving. Many dual process models were designed to classify the “type” of thinking process or a form of cognitive processing. In some sense, the “processes” in dual process theories are characterized by the type of mechanism of operation or the type of output they produced. Here, I instead characterize and differentiate the modes of thinking by the receptivity of different functional units in the brain to input during problem solving.

This, however, raises a different question of the relationship between these attentional modes and conscious vs. unconscious thinking. It is clear that both the conscious and unconscious are involved in problem solving, as well as in RWPS. Here, I claim that a problem being handled is, at any given point in time, in either a focused mode or in a defocused mode. When in the focused mode, problem solving primarily proceeds in a manner that is available for conscious deliberation. More specifically, problem space elements and representations are tightly managed and plans and strategies are available in the working memory and consciously accessible. There are, however, secondary unconscious operations in the focused modes that includes targeted memory retrieval and heuristic-based searches. In the defocused mode, the problem is primarily managed in an unconscious way. The problem space elements are broken apart and loosely managed by various mechanisms that do not allow for conscious deliberation. That said, it is possible that some problem parameters remain accessible. For example, it is possible that certain goal information is still maintained consciously. It is also possible that indexes to all the problems being considered by the solver are maintained and available to conscious awareness.

### 5.2. RWPS model

**Figure 1:**
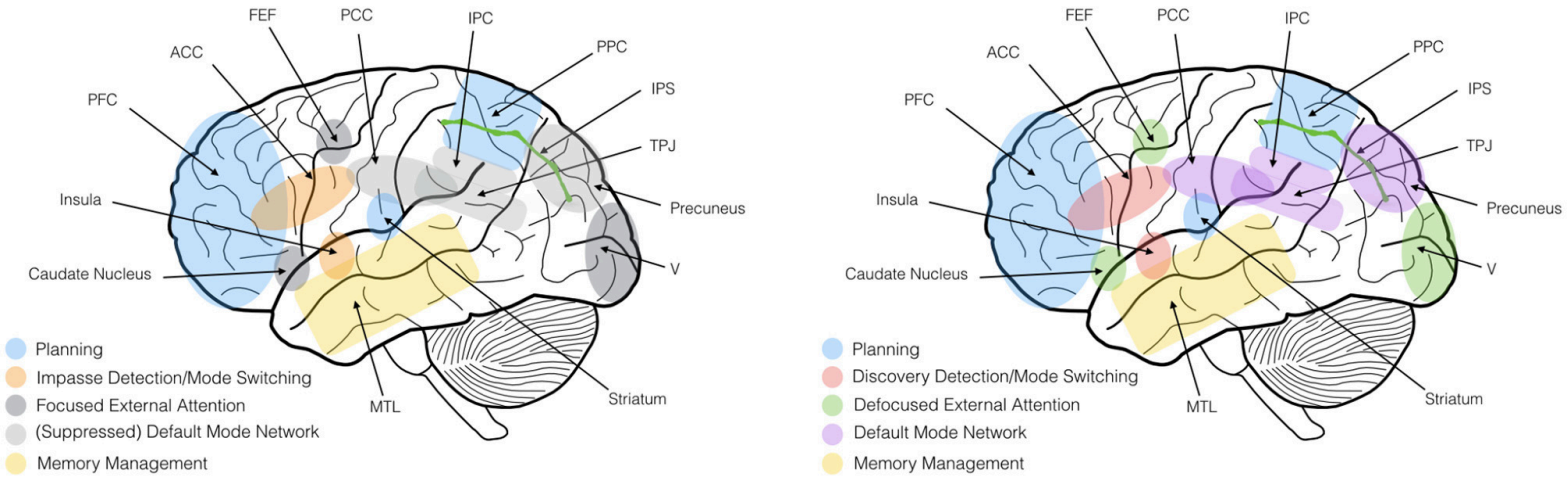
Summary of neural activations during focused problem-solving **(Left)** and defocused problem-solving **(Right)**. During defocused problem-solving, the salience network (insula and ACC) coordinates the switching of several networks into a defocused attention mode that permits the reception of a more varied set of stimuli and interpretations via both the internally-guided networks (default mode network DMN) and externally guided networks (Attention). PFC, prefrontal cortex; ACC, anterior cingulate cortex; PCC, posterior cingulate cortex; IPC, inferior parietal cortex; PPC, posterior parietal cortex; IPS, intra-parietal sulcus; TPJ, temporoparietal junction; MTL, medial temporal lobe; FEF, frontal eye field.

**Figure 2:**
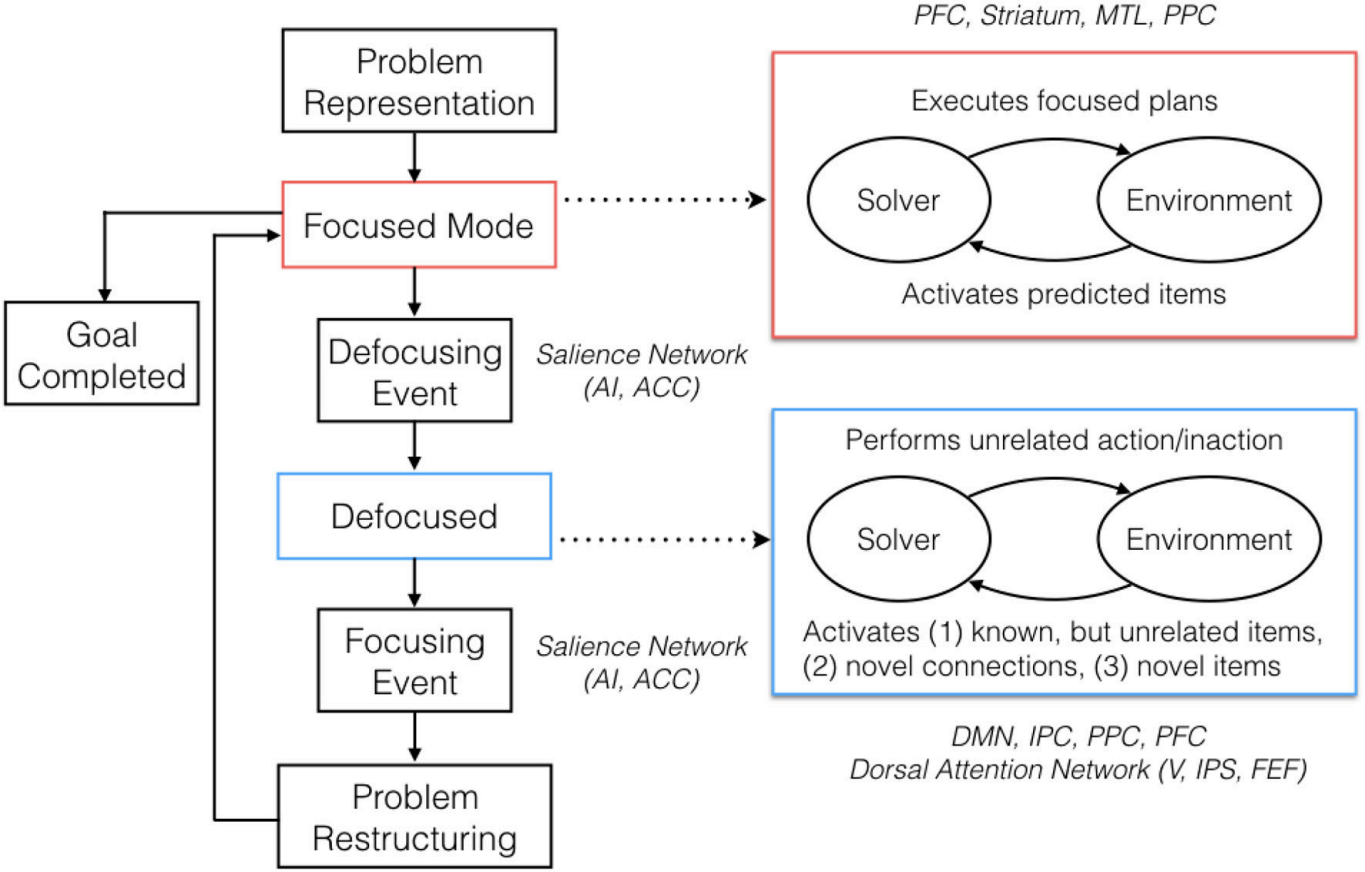
Proposed Model for Real World Problem Solving (RWPS). The corresponding neural correlates are shown in italics. During problem-solving, an initial problem representation is formed based on prior knowledge and available perceptual information. The problem-solving then proceeds in a focused, goal-directed mode until the goal is achieved or a defocusing event (e.g., impasse or distraction) occurs. During focused mode operation, the solver interacts with the environment in directed manner, executing focused plans, and allowing for predicted items to be activated by the environment. When a defocusing event occurs, the problem-solving then switches into a defocused mode until a focusing event (e.g., discovery) occurs. In defocused mode, the solver performs actions unrelated to the problem (or is inactive) and is receptive to a set of environmental triggers that activate novel aspects using the three mechanisms discussed in this paper. When a focusing event occurs, the diffused problem elements cohere into a restructured representation and problem-solving returns into a focused mode.

Returning to Ollinger's model for insight problem solving, it now becomes readily apparent how this model can be modified to incorporate environmental effects as well as generalizing the notion of intervening events beyond that of impasses. I propose a theory for RWPS that begins with standard analytical problem-solving process (See Figures 1, 2).

#### 5.2.1. Focused problem solving mode

Initially, both prior knowledge and perceptual entities help guide the creation of problem representations in working memory. Prior optimal or rewarding solution strategies are obtained from LTM and encoded in the working memory as well. This process is largely analytical and the solver interacts with their environment through focused plan or idea execution, targeted observation of prescribed entities, and estimating prediction error of these known entities. More specifically, when a problem is presented, the problem representations are activated and populated into working memory in the PFC, possibly in structured representations along convergence zones. The PFC along with the Striatum and the MTL together attempt at retrieving an optimal or previously rewarded solution strategy from long term memory. If successfully retrieved, the solution strategy is encoded into the PPC as a mental template, which then guides relevant motor control regions to execute the plan.

#### 5.2.2. Defocusing event-triggered mode switching

The search and solve strategy then proceeds analytically until a “defocusing event” is encountered. The salience network (AI and ACC) monitor for conflicts and attempt to detect any such events in the problem-solving process. As long as no conflicts are detected, the salience network focuses on recruiting networks to achieve goals and suppresses the DMN (Beaty et al., 2016). If the plan execution or retrieval of the solution strategy fails, then a defocusing event is detected and the salience network performs mode switching. The salience network dynamically switches from the focused problem-solving mode to a defocused problem-solving mode (Menon, 2015). Ollinger's current model does not account for other defocusing events beyond an impasse, but it is not inconceivable that there could be other such events triggered by external stimuli (e.g., distraction or an affective event) or by internal stimuli (e.g., mind wandering).

#### 5.2.3. Defocused problem solving mode

In defocused mode, the problem is operated on by mechanisms that allow for the generation and testing of novel ideas. Several large-scale brain networks are recruited to explore and generate new ideas. The search for novel ideas is facilitated by generally defocused attention, which in turn allows for creative idea generation from both internal as well as external sources. The salience network switches operations from defocused event detection to focused event or discovery detection, whereby for example, environmental events or ideas that are deemed interesting can be detected. During this idea exploration phase, internally, the DMN is no longer suppressed and attempts to generate new ideas for problem-solving. It is known that the IPC is involved in the generation of new ideas (Benedek et al., 2014) and together with the PPC in coupling different information together (Simone Sandkühler, 2008; Stocco et al., 2012). Beaty et al. (2016) have proposed that even this internal idea-generation process can be goal directed, thereby allowing for a closer working relationship between the CEN and the DMN. They point to neuroimaging evidence that support the possibility that the executive control network (comprising the lateral prefrontal and inferior parietal regions) can constrain and direct the DMN in its process of generating ideas to meet task-specific goals via top down monitoring and executive control (Beaty et al., 2016). The control network is believed to maintain an “internal train of thought” by keeping the task goal activated, thereby allowing for strategic and goal-congruent searches for ideas. Moreover, they suggest that the extent of CEN involvement in the DMN idea-generation may depend on the extent to which the creative task is constrained. In the RWPS setting, I would suspect that the internal search for creative solutions is not entirely unconstrained, even in the defocused mode. Instead, the solver is working on a specified problem and thus, must maintain the problem-thread while searching for solutions. Moreover, self-generated ideas must be evaluated against the problem parameters and thereby might need some top-down processing. This would suggest that in such circumstances, we would expect to see an increased involvement of the CEN in constraining the DMN.

On the external front, several mechanisms are operating in this defocused mode. Of particular note are the dorsal attention network, composed of the visual cortex (V), IPS and the frontal eye field (FEF) along with the precuneus and the caudate nucleus allow for partial cues to be considered. The MTL receives synthesized cue and contextual information and populates the WM in the PFC with a potentially expanded set of information that might be relevant for problem-solving. The precuneus, dlPFC and PPC together trigger the activation and use of a heuristic prototype based on an event in the environment. The caudate nucleus facilitates information routing between the PFC and PPC and is involved in learning and skill acquisition.

#### 5.2.4. Focusing event-triggered mode switching

The problem's life in this defocused mode continues until a focusing event occurs, which could be triggered by either external (e.g., notification of impending deadline, discovery of a novel property in the environment) or internal items (e.g., goal completion, discovery of novel association or updated relevancy of a previously irrelevant item). As noted earlier, an internal train of thought may be maintained that facilitates top-down evaluation of ideas and tracking of these triggers (Beaty et al., 2016). The salience network switches various networks back to the focused problem-solving mode, but not without the potential for problem restructuring. As noted earlier, problem space elements are maintained somewhat loosely in the defocused mode. Thus, upon a focusing event, a set or subset of these elements cohere into a tight (restructured) representation suitable for focused mode problem solving. The process then repeats itself until the goal has been achieved.

### 5.3. Model predictions

#### 5.3.1. Single-mode operation

The proposed RWPS model provides several interesting hypotheses, which I discuss next. First, the model assumes that any given problem being worked on is in one mode or another, but not both. Thus, the model predicts that there cannot be focused plan execution on a problem that is in defocused mode. The corollary prediction is that novel perceptual cues (as those discussed in section 4) cannot help the solver when in focused mode. The corollary prediction, presumably has some support from the inattentional blindness literature. Inattentional blindness is when perceptual cues are not noticed during a task (e.g., counting the number of basketball passes between several people, but not noticing a gorilla in the scene) (Simons and Chabris, 1999). It is possible that during focused problem solving, that external and internally generated novel ideas are simply not considered for problem solving. I am not claiming that these perceptual cues are always ignored, but that they are not considered within the problem. Sometimes external cues (like distracting occurrences) can serve as defocusing events, but the model predicts that the actual content of these cues are not themselves useful for solving the specific problem at hand.

When comparing dual-process models Sowden et al. (2015) discuss shifting from one type of thinking to another and explore how this shift relates to creativity. In this regard, they weigh the pros and cons of serial vs. parallel shifts. In dual-process models that suggest serial shifts, it is necessary to disengage one type of thought prior to engaging the other or to shift along a continuum. Whereas, in models that suggest parallel shifts, each of the thinking types can operate in parallel. Per this construction, the proposed RWPS model is serial, however, not quite in the same sense. As noted earlier, the RWPS model is not a dual-process model in the same sense as other dual process model. Instead, here, the thrust is on when the brain is receptive or not receptive to certain kinds of internal and external stimuli that can influence problem solving. Thus, while the modes may be serial with respect to a certain problem, it does not preclude the possibility of serial and parallel thinking processes that might be involved within these modes.

#### 5.3.2. Event-driven transitions

The model requires an event (defocusing or focusing) to transition from one mode to another. After all why else would a problem that is successfully being resolved in the focused mode (toward completion) need to necessarily be transferred to defocused mode? These events are interpreted as conflicts in the brain and therefore the mode-switching is enabled by the saliency network and the ACC. Thus, the model predicts that there can be no transition from one mode to another without an event. This is a bit circular, as an event is really what triggers the transition in the first place. But, here I am suggesting that an external or internal cue triggered event is what drives the transition, and that transitions cannot happen organically without such an event. In some sense, the argument is that the transition is discontinuous, rather than a smooth one. Mind-wandering is good example of when we might drift into defocused mode, which I suggest is an example of an internally driven event caused by an alternative thought that takes attention away from the problem.

A model assumption underlying RWPS is that events such as impasses have a similar effect to other events such as distraction or mind wandering. Thus, it is crucial to be able to establish that there exists of class of such events and they have a shared effect on RWPS, which is to switch attentional modes.

#### 5.3.3. Focused mode completion

The model also predicts that problems cannot be solved (i.e., completed) within the defocused mode. A problem can be considered solved when a goal is reached. However, if a goal is reached and a problem is completed in the defocused mode, then there must have not been any converging event or coherence of problem elements. While it is possible that the solver arbitrarily arrived at the goal in a diffused problem space and without conscious awareness of completing the task or even any converging event or problem recompiling, it appears somewhat unlikely. It is true that there are many tasks that we complete without actively thinking about it. We do not think about what foot to place in front of another while walking, but this is not an instance of problem solving. Instead, this is an instance of unconscious task completion.

#### 5.3.4. Restructuring required

The model predicts that a problem cannot return to a focused mode without some amount of restructuring. That is, once defocused, the problem is essentially never the same again. The problem elements begin interacting with other internally and externally-generated items, which in turn become absorbed into the problem representation. This prediction can potentially be tested by establishing some preliminary knowledge, and then showing one group of subjects the same knowledge as before, while showing the another group of subjects different stimuli. If the model's predictions hold, the problem representation will be restructured in some way for both groups.

There are numerous other such predictions, which are beyond the scope of this paper. One of the biggest challenges then becomes evaluating the model to set up suitable experiments aimed at testing the predictions and falsifying the theory, which I address next.

## 6. Experimental challenges and paradigms

One of challenges in evaluating the RWPS is that real world factors cannot realistically be accounted for and sufficiently controlled within a laboratory environment. So, how can one controllably test the various predictions and model assumptions of “real world” problem solving, especially given that by definition RWPS involves the external environment and unconscious processing? At the expense of ecological validity, much of insight problem solving research has employed an experimental paradigm that involves providing participants single instances of suitably difficult problems as stimuli and observing various physiological, neurological and behavioral measures. In addition, through verbal protocols, experimenters have been able to capture subjective accounts and problem solving processes that are available to the participants' conscious. These experiments have been made more sophisticated through the use of timed-hints and/or distractions. One challenge with this paradigm has been the selection of a suitable set of appropriately difficult problems. The classic insight problems (e.g., Nine-dot, eight-coin) can be quite difficult, requiring complicated problem solving processes, and also might not generalize to other problems or real world problems. Some in the insight research community have moved in the direction of verbal tasks (e.g., riddles, anagrams, matchstick rebus, remote associates tasks, and compound remote associates tasks). Unfortunately, these puzzles, while providing a great degree of controllability and repeatability, are even less realistic. These problems are not entirely congruent with the kinds of problems that humans are solving every day.

The other challenge with insight experiments is the selection of appropriate performance and process tracking measures. Most commonly, insight researchers use measures such as time to solution, probability of finding solution, and the like for performance measures. For process tracking, verbal protocols, coded solution attempts, and eye tracking are increasingly common. In neuroscientific studies of insight various neurological measures using functional magnetic resonance imaging (fMRI), electroencephalography (EEGs), transcranial direct current stimulation (tDCS), and transcranial magnetic stimulation (tMS) are popular and allow for spatially and temporally localizing an insight event.

Thus, the challenge for RWPS is two-fold: (1) selection of stimuli (real world problems) that are generalizable, and (2) selection of measures (or a set of measures) that can capture key aspects of the problem solving process. Unfortunately, these two challenges are somewhat at odds with each other. While fMRI and various neuroscientific measures can capture the problem solving process in real time, it is practically difficult to provide participants a realistic scenario while they are laying flat on their back in an fMRI machine and allowed to move nothing more than a finger. To begin addressing this conundrum, I suggest returning to object manipulation problems (not all that different from those originally introduced by Maier and Duncker nearly a century ago), but using modern computing and user-interface technologies.

One pseudo-realistic approach is to generate challenging object manipulation problems in Virtual Reality (VR). VR has been used to describe 3-D environment displays that allows participants to interact with artificially projected, but experientially realistic scenarios. It has been suggested that virtual environments (VE) invoke the same cognitive modules as real equivalent environmental experience (Foreman, 2010). Crucially, since VE's can be scaled and designed as desired, they provide a unique opportunity to study pseudo-RWPS. However, a VR-based research approach has its limitations, one of which is that it is nearly impossible to track participant progress through a virtual problem using popular neuroscientific measures such as fMRI because of the limited mobility of connected participants.

Most of the studies cited in this paper utilized an fMRI-based approach in conjunction with a verbal or visual task involving problem-solving or creative thinking. Very few, if any, studies involved the use physical manipulation, and those physical manipulations were restricted to limited finger movements. Thus, another pseudo-realistic approach is allowing subjects to teleoperate robotic arms and legs from inside the fMRI machine. This paradigm has seen limited usage in psychology and robotics, in studies focused on Human-Robot interaction (Loth et al., 2015). It could be an invaluable tool in studying real-time dynamic problem-solving through the control of a robotic arm. In this paradigm a problem solving task involving physical manipulation is presented to the subject via the cameras of a robot. The subject (in an fMRI) can push buttons to operate the robot and interact with its environment. While the subjects are not themselves moving, they can still manipulate objects in the real world. What makes this paradigm all the more interesting is that the subject's manipulation-capabilities can be systematically controlled. Thus, for a particular problem, different robotic perceptual and manipulation capabilities can be exposed, allowing researchers to study solver-problem dynamics in a new way. For example, even simple manipulation problems (e.g., re-arranging and stacking blocks on a table) can be turned into challenging problems when the robotic movements are restricted. Here, the problem space restrictions are imposed not necessarily on the underlying problem, but on the solver's own capabilities. Problems of this nature, given their simple structure, may enable studying everyday practical creativity without the burden of devising complex creative puzzles. Crucial to note, both these pseudo-realistic paradigms proposed demonstrate a tight interplay between the solver's own capabilities and their environment.

## 7. Conclusion

While the neural basis for problem-solving, creativity and insight have been studied extensively in the past, there is still a lack of understanding of the role of the environment in informing the problem-solving process. Current research has primarily focused on internally-guided mental processes for idea generation and evaluation. However, the type of real world problem-solving (RWPS) that is often considered a hallmark of human intelligence has involved both a dynamic interaction with the environment and the ability to handle intervening and interrupting events. In this paper, I have attempted to synthesize the literature into a unified theory of RWPS, with a specific focus on ways in which the environment can help problem-solve and the key neural networks involved in processing and utilizing relevant and useful environmental information. Understanding the neural basis for RWPS will allow us to be better situated to solve difficult problems. Moreover, for researchers in computer science and artificial intelligence, clues into the neural underpinnings of the computations taking place during creative RWPS, can inform the design the next generation of helper and exploration robots which need these capabilities in order to be resourceful and resilient in the open-world.

## Author contributions

The author confirms being the sole contributor of this work and approved it for publication.

### Conflict of interest statement

The author declares that the research was conducted in the absence of any commercial or financial relationships that could be construed as a potential conflict of interest.

## Footnotes

^1^My intention is not to ignore the benefits of a concentrated internal thought process which likely occurred as well, but merely to acknowledge the possibility that the environment might have also helped.

^2^The research in insight does extensively use “hints” which are, arguably, a form of external influence. But these hints are highly targeted and might not be available in this explicit form when solving problems in the real world.

^3^The accuracy of these accounts has been placed in doubt. They often are recounted years later, with inaccuracies, and embellished for dramatic effect.

^4^I use the term “agent” to refer to the problem-solver. The term agent is more general than “creature” or “person” or “you" and is intentionally selected to broadly reference humans, animals as well as artificial agents. I also selectively use the term “solver.”
